# Immunoneutralization of TGF*β*1 Improves Skeletal Muscle Regeneration: Effects on Myoblast Differentiation and Glycosaminoglycan Content

**DOI:** 10.1155/2009/659372

**Published:** 2009-05-10

**Authors:** M. Zimowska, A. Duchesnay, P. Dragun, A. Oberbek, J. Moraczewski, I. Martelly

**Affiliations:** ^1^Department of Cytology, Institute of Zoology, Faculty of Biology, University of Warsaw, Warsaw, Poland; ^2^Laboratoire de Recherche sur la Croissance Cellulaire, la Régénération et la Réparation Tissulaires (CRRET), CNRS UMR7149, Faculté de Sciences et technologie, Université Paris EST, Paris 12 Val de Marne, 61 avenue du Général de Gaulle, 94010 Créteil, France

## Abstract

When injured by crushing, the repair of the slow-twitch soleus rat muscle, unlike the fast-twitch EDL, is associated with fibrosis. As TGF*β*1, whose activity can be controlled by glycosaminoglycans (GAG), plays a major role in fibrosis, we hypothesized that levels of TGF*β*1 and GAG contents could account for this differential quality of regeneration. Here we show that the regeneration of the soleus was accompanied by elevated and more sustained TGF*β*1 level than in the EDL. Neutralization of TGF*β*1 effects by antibodies to TGF*β*1 or its receptor TGF*β*-R1 improved muscle repair, especially of the soleus muscle, increased in vitro growth of myoblasts, and accelerated their differentiation. These processes were accompanied by alterations of GAG contents. These results indicate that the control of TGF*β*1 activity is important to improve regeneration of injured muscle and accelerate myoblast differentiation, in part through changes in GAG composition of muscle cell environment.

## 1. Introduction

Skeletal muscle has a great ability to regenerate in response to injury or disease [1, 2]. The regeneration process is attributed to mononucleated cells found in adult skeletal muscles located between the basal lamina and the plasmalemma of muscle fibers. Recent work has shown that these so-called satellite cells are a heterogeneous population comprising stem cells and committed progenitor cells (reviewed in [3–6]). Stimulated by damage to the muscle fiber, satellite cells are activated to enter the cell cycle; they fuse either together or with myofibers in order to restore muscle architecture and function with more or less efficiency [2, 7, 8].

Most muscles are a mosaic of fast and slow fibers [9, 10]. Due to their composition, the slow-twitch muscle soleus and the fast-twitch muscle *Extensor digitorum longus* (EDLs) are widely used as models because they are composed in majority or almost totally of slow or fast type fibers reciprocally, depending on animal species. Studies of the regenerative response of these two muscles to injury, induced, for example, by a microlesion [11] or an anesthetic injection [12, 13], have found differences in the way these two types of muscle get repaired. This is particularly true in a whole muscle crush model in rat where the soleus muscle undergoes fibrosis in contrast to the EDL muscle that regenerates correctly [14].

Intrinsic properties of satellite cells involved in muscle repair might contribute to the differential repair processes of fast and slow skeletal muscles in rats. Several studies, including our previous works [13, 15–17], have reported that myoblasts isolated from fast or slow muscles and grown in vitro display some differential growth and differentiation properties. The differences of myoblasts from fast and slow muscles are maintained independently of the phenotypic transition of muscle fibers induced by an adequate chronic electrical stimulation.

Cytokine and growth factor activities could be proposed to account for the differential features of regenerating slow and fast skeletal muscles. Among growth factors known to modulate the regeneration process, those belonging to the transforming growth factor beta (TGF*β*) family seem to play specific roles in muscle development and after injury [18]. Works in the 1980s and after have shown that TGF*β* negatively regulates muscle cell proliferation and differentiation [19]. This cytokine can also influence muscle fiber type patterning in regenerating muscles after injury [20]. In addition, TGF*β* family members are well known to play a major role in fibrosis development and scar formation since they stimulate extracellular matrix (ECM) production, modulate the expression of ECM-degrading enzymes and proteinase inhibitors [21, 22]. In the C2C12 myoblast cell line, TGF*β*1 can stimulate its own synthesis in an autocrine manner and triggers cells to differentiate into myofibroblasts in injured muscle [23]. TGF*β*1 is also connected with fibrosis during congenital muscular dystrophy and Duchenne's dystrophy [24–26]. Thus, this cytokine initiates a fibrotic cascade in skeletal muscles that needs to be hindered [24, 27, 28] and reduction of TGF*β*1 expression or activity would appear promising for improving muscle repair, as suggested by earlier studies [25, 29].

Differences in matrix component environment could also be evoked to account for muscle regeneration ability in particular in the case of the whole muscle crush model. Muscle environment has a profound effect on the regenerative capacity of resident muscle precursor cells and implanted cells [30, 31]. Among ECM components and membrane-associated components that contribute to cellular environment, proteoglycans (PGs) have been implicated in numerous physiological and pathological processes such as enzyme regulation, cellular adhesion, growth, migration, and differentiation [32, 33]. The effect of PG is mainly due to the glycosaminoglycan (GAG) moiety of these molecules, principally the heparan sulphate (HS) and the dermatan sulphate/chondroitine sulphate (CS/DS) (see [34, 35] for reviews). In particular, HS plays a prominent role in skeletal muscle development and physiology [36]. We have demonstrated that HS is increased during differentiating myoblast [37]. ECM composes a reservoir for growth factors including TGF*β* [38, 39]. Among PGs that belong to muscle environment, some of them such as decorin, biglycan, and betaglycan have been implicated in the regulation of TGF*β* bioavailability in skeletal muscle, modulating myogenesis progression [26, 40]. It is generally believed that TGF*β* signaling is initiated by its binding to the proteoglycan betaglycan and then to the TGF*β* receptors type II and I heterodimer from where a signal is transduced [41–43]. Although TGF*β* is known to bind to betaglycan core protein [44], modifications in GAG moiety of this PG modulate TGF*β* binding to TGF*β* receptors and modulate downstream signalling [45]. Thus, the acquaintance with GAG content and composition after tissue injury may be of particular importance in the understanding of processes involved in tissue repair. The balance between secreted cytokines such as TGF*β* and GAG network may represent a homeostatic mechanism aimed at controlling the skeletal muscle repair process.

The main hypothesis presently explored was that TGF*β*1 level could at least partially account for the differential regeneration abilities of soleus and EDL muscles through modulating myoblast differentiation and GAG cellular environment. An impairment of TGF*β* activity was achieved using injection of anti-TGF*β*1 or anti-TGF*β* receptor I (T*β*RI) antibodies into crushed soleus or EDL muscles in vivo or by treating primary cultures of myoblasts isolated from both muscles with these antibodies. The effects of these treatments were analyzed on muscle regeneration and myoblast differentiation. Attention was also focused on GAGs in the environment of satellite cells and in regenerating muscles. Taken together, results suggested that TGF*β*1 and GAGs interplay in regulating muscle repair and myoblast differentiation.

## 2. Materials and Methods

### 2.1. Materials

Dulbecco modified essential medium (DMEM), foetal bovine serum (FBS), and horse serum were from Gibco, Invitrogen Life technology. Primary and secondary antibodies were from Santa Cruz Biotech. PVDF membrane and immunoblotting detection reagents were from Roche. Reagents for ELISA assay were from R&D, BD Biosciences. Other reagents were purchased from Sigma.

### 2.2. Surgical Procedure and Regeneration Experiments

All procedures involving animals were approved by the Ethics Committee in the Care and use of Animals (192/2002). The regeneration of soleus and EDL muscles was induced in 3-month-old male Wistar rats according to [14]. In brief, rats were anaesthetized with an intraperitoneal injection of ketamine (6 mg/kg) and xylasine (60 mg/kg) mixture and the muscle was exposed. Tendons were kept in place but motor nerve was cut at the muscle surface. Then muscle was crushed from tendon to tendon with a Pean haemostatic forceps. The muscle was then put back in its bed. After skin closure, animals were allowed to recover and were returned to their cage with food and drink *at libitum*. This procedure ensured a good reproducibility of the regeneration process and induced an exhaustive myolysis of all fibers thus validating further biochemical analysis on injured muscles. Antibodies against TGF*β*1 or TGF*β*-receptor I (T*β*RI) were injected just after crush (100 *μ*g per muscle in 50 *μ*L) into the muscles referred to as treated muscles. At different days after injury, animals were euthanasized in *CO*_2_ and the regenerating muscles of each animal were removed and weighed. Muscles were then either directly used for biochemical investigations or freezed in isopentane precooled in liquid nitrogen, then stored at −80°C pending histological or biochemical studies. Three rats were used for each muscle type and the experiment was repeated 3 times. Contralateral intact muscles were also taken for comparison to injured muscles.

### 2.3. Histological Analysis of Regenerated Muscle

Transversal cross-sections of control or treated soleus and EDL muscles were cut (10 *μ*m thick) on a cryostat (Microm, Germany) and stained with the Gomori Trichrome technique for histological examination.

### 2.4. Myoblast Primary Cultures

Satellite cells were dissociated with pronase from soleus or EDL muscles of 3 months old Wistar male rats, as previously described [16]. Cells were seeded (2 000 cells/c*m* ^2^) on dishes coated with 0.1% gelatin and grown continuously in DMEM containing 10% fetal bovine serum and 10% horse serum in 5% *CO*_2_ at 37°C. At days 4, 6, 8, 10, 12, or 14 cellular growth was measured by counting the cells after trypsinization medium was collected and cells were homogenized for immunoblots or for GAG measurements. Histological aspects of the cultures were assessed by Hoffman contrast observations (Nikon microscope). Some cultures were stained with the Giemsa-May-Grünwald technique for myotube analysis and fusion index determination. The size of myotubes was evaluated by counting the number of nuclei found in each myotube. The fusion index represents the percentage of nuclei found in myotubes divided by the total number of nuclei in the culture. These counts were performed on 10 representative microscopic fields of each plate belonging to several cultures.

### 2.5. Cell Treatments

In some experiments, myoblast cultures were treated either with anti-TGF*β*1 or anti-T*β*RI antibodies. According to preliminary experiments performed to determine the optimum concentration and frequency of treatment with antibodies to be used, soleus or EDL myoblast cultures were treated at day 4 and then every 2 days along with medium change with 1 *μ*g/mL anti-TGF*β*1 antibody. Either 1.5 *μ*g/mL or 1.0 *μ*g/mL of anti-T*β*RI was used on soleus and EDL myoblasts, respectively.

### 2.6. Immunoblot Analysis

Experiments were performed for control or treated regenerated muscles and cell cultures. Regenerated muscle in vivo or cells grown in vitro were lysed in ice-cold buffer containing 20 mM Tris-HCl, 5 mM EGTA, 5 mM EDTA, 150 mM KCL, 1% Nonidet, 0.5% sodium deoxycholate, 0.01% leupeptin, 0.5 mM PMSF, and 10 mM *β*-mercaptoethanol at pH 7.5. All operations were performed on ice. Protein concentration was quantitatively determined using the Bradford Biorad Protein assay. Twenty *μ*g of protein in Laemmli sample buffer were loaded onto a sodium dodecyl sulfate 10% acrylamide gel, transferred to PVDF membrane, and incubated with polyclonal antibodies raised against TGF*β*1 or T*β*RI or Fibronectin at final dilution 1 : 200 (overnight, 4°C). The blots were then incubated with peroxidase-conjugated antirabbit antibodies (1 : 2000, 1.5 hours, room temperature). The loading of gels was routinely controlled using anti-*α*-tubuline [46]. The immunoblots were visualized by chemiluminescence and exposed to film (Kodak). An image of the gel was captured using the GelDoc2000 scanner and protein bands were analyzed using Quantity One program (BIORAD) and graphed using Excel. Each experiment was performed 3 times and the results were expressed in percentage as mean ± SE.

### 2.7. ELISA Measurement of TGF*β*1

Immunoassays were performed with extracts from control soleus or EDL regenerating muscles. Muscles were homogenized in lysis buffer; protein concentrations were determined using the Bradford Biorad Assay. One hundred *μ*L of samples containing 20 *μ*g of total protein were assayed according to the manufacturer procedure. Duplicate measurements for each standard and samples were performed.

### 2.8. Sulphated GAG Measurement

Total sulphated GAGs were measured as described in Barbosa et al. 2003 [47]. In brief, homogenates from cellular layer or muscle fragments were performed in phosphate buffer (*K*_2_HP*O*_4_, 50 mM, pH 8.0). Cellular extracts were then treated with 50 *μ*g/mL proteinase K in phosphate buffer at 56°C overnight. Heating the preparation 10 minutes at 90°C then inactivating proteinase K, at this step, the amount of DNA in aliquot samples was determined by 4,6-diamino-2-phenylindole (DAPI) assay using salmon sperm DNA as standard [48]. Digested tissues were then treated with DNAse (Qiagen, France) used at 7.5 units/100 mg fresh weight overnight at 37°C in order to eliminate interfering DNA. These preparations were used for sulphated GAG quantitation.

Total sulphated GAGs and HS GAG species were quantitated using the method based on dimethylmethylene blue co-precipitation with GAGs according to [47]. HS was quantitated after treatment of total GAG preparations with chondroïtinase ABC (20 mU/100 *μ*L, 2 hours, Sigma).

### 2.9. Statistical Analysis

Each experiment was repeated at least three times. Data are expressed as mean ± standard error (SE). Statistical significance was determined using a Student's *t*-test (GraphPad Software, San Diego, Calif, USA).

## 3. Results

### 3.1. Differential Characteristics of Regenerating Soleus and EDL Muscles after Crush

**Figure 1:**
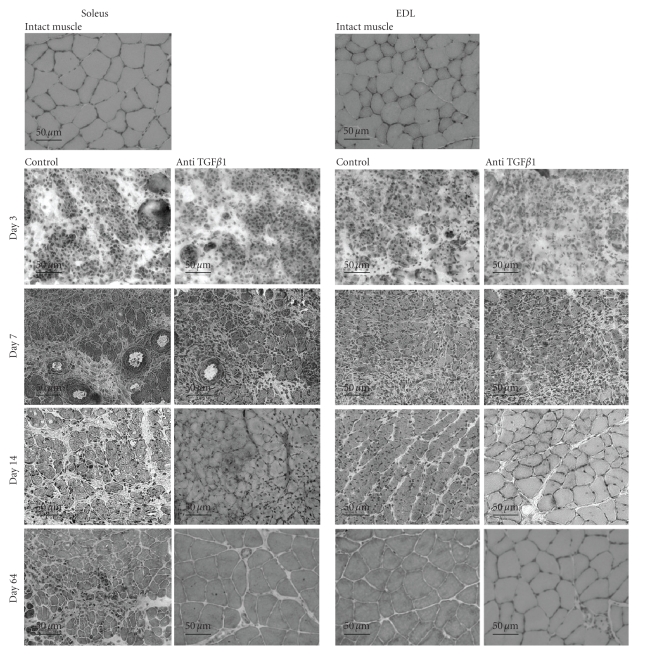
*Histological aspects of regenerating soleus and EDL muscles.* Transversal cryosections of intact muscles, control muscles, or muscles that were injected with anti-TGF*β*1 antibody were performed at the indicated days after crush. Sections (10 *μ*m) were stained with Gomori Trichrome as described in the Materials and Methods Section. Bars: 50 *μ*m.

**Figure 2:**
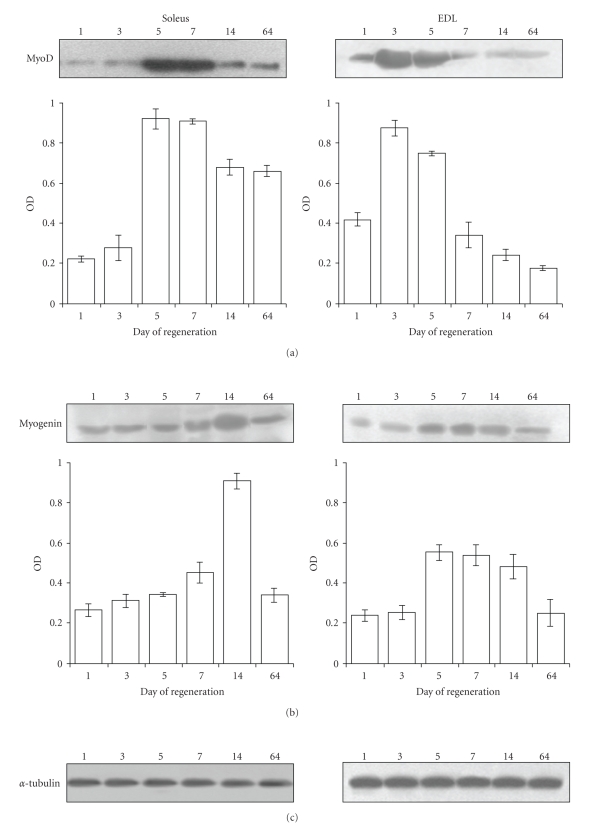
*Pattern of MyoD and myogenin proteins in regenerating EDL and Soleus muscles*. Representative immunoblots of (a) MyoD and (b) myogenin performed on extracts from regenerating soleus and EDL muscles at the indicated days after crush are shown. (c) Alpha**tubulin was routinely used as control of loading. Band densities of membranes from 3 different samples were scanned as described in Materials and Methods and mean values ± SE are shown as OD arbitrary units.

Histology of soleus and EDL muscles in the course of crush-induced regeneration is shown on Figure 1. After crush, a complete muscle fiber myolysis occurs in both muscles, that is, almost achieved at day 3 (Figure 1 Control pictures). At day 7, numerous small fibers can be observed in both soleus and EDL muscles. However, whereas the EDL muscle was regenerated correctly and showed a well-structured muscle at days 14 and 64 after crush, the soleus showed heterogeneous fiber size at day 14 after crush. Regeneration did not progress further and fibrosis, already obvious at day 14, remained at day 64. Both types of muscles also differed by muscle regulatory factor (MRF) protein patterns. Indeed, MRFs were activated earlier in regenerating EDL than in soleus muscle. In both muscles, levels of MyoD and myogenin were successively increased. However, in soleus muscle, MyoD peaked at days 5–7 and myogenin at day 14, whereas in EDL muscles these MRFs peaked earlier, MyoD being at its maximum at day 3 and myogenin at day 7 (Figure 2).

**Figure 3:**
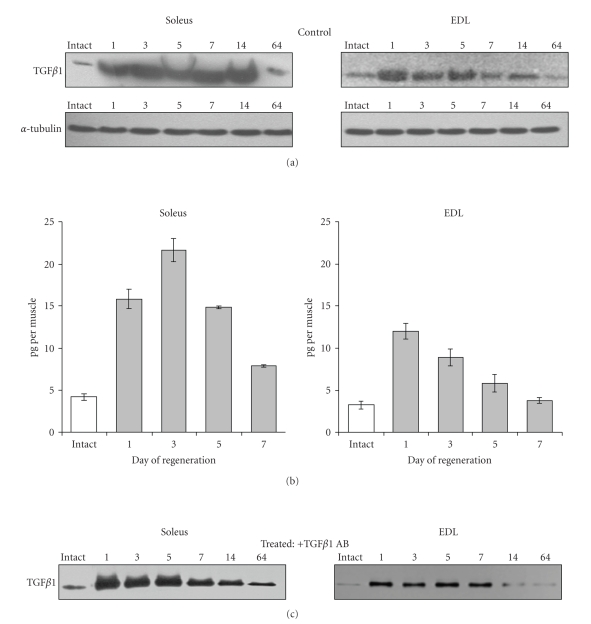
*TGF* *β* *1 protein levels during soleus and EDL muscle regeneration*. (a) Representative immunoblots of TGF*β*1 performed on extracts from control untreated soleus and EDL muscles taken at the indicated days after crush. Alphatubulin was used as control of loading. (b) TGF*β*1 protein amount measured by ELISA during the first week after crush in untreated regenerating muscles (control). Levels of TGF*β*1 are shown as pg per muscle fresh weight. In intact soleus and EDL muscles 4.19 pg per muscle and 3.24 pg per muscle were found, respectively. The data represent mean ± SE of 3 independent determinations each of them including 3 animals. All values found in regenerating muscles were statistically different from intact muscles (*P* < .05) except for EDL day 7. (c) Representative Immunoblots of TGF*β*1 in extracts from muscles injected just after crush with anti-TGF*β*1 antibody and taken at the indicated days after crush.

Immunoblot analysis of TGF*β*1 performed with extracts prepared from regenerating muscles (Figure 3(a)) revealed that in soleus muscle TGF*β*1 increased at day 1 after crush and remained at a high level up to 14 days. At day 64 after crush, TGF*β*1 level was still slightly higher than in intact muscle. In EDL regenerating muscle, TGF*β*1 amount increased at day 1 then diminished to the control level as early as day 7 after crush. Measurements of TGF*β*1 by ELISA technique correlated with immunoblotting evaluation at least for the first 7 days after crush. (Figure 3(b)). The amount of TGF*β*1 protein in intact soleus muscle was 4.19 pg ± 0.39. In this muscle, it increased 3 to 4 times at days 1 and 3 after crush to reach more than 21 pg per muscle at day 3; then, it decreased at day 7, when it still remained about twice higher than in control muscle. In the EDL intact muscle, TGF*β*1 protein amount measured by ELISA was 3.24 pg ± 0.44. It increased to about 12 pg/muscle at day 1, then regularly decreased reaching the control level at day 7.

Thus, the pattern of TGF*β*1 protein level whatever its origin (inflammatory cells or muscle cells) is different in soleus and EDL regenerating muscles. It remained at a higher level a much longer time in soleus than in EDL muscle.

### 3.2. TGF*β*1 Antibody Improves Skeletal Muscle Regeneration

Anti-TGF*β*1 antibodies were injected into the injured muscle after crush to further validate the hypothesis that sustained TGF*β*1 action would explain the presence of fibrosis in regenerating soleus. The presence of anti-TGF*β*1 improved the structure of regenerating muscles (on Figure 1 see “anti-TGF*β*1” compared to “controls”). Muscle regeneration improvement was especially remarkable in the case of soleus muscle. The diameters of fibers were larger and more homogenous in size. Treatment of muscles of both types with anti-TGF*β*1 antibody allowed the restoration of muscular architecture similar to an intact muscle at day 64.

It was not possible to quantitate TGF*β*1 by ELISA technique in the anti-TGF*β*1-treated muscles because of the presence of antibodies in the extract that would interfere with the test but immunoblot showed that the treatment of soleus or EDL muscles with anti-TGF*β*1 antibody reduced the amount of TGF*β*1 protein compared to untreated regenerating muscles (Figure 3(c)). Interestingly, TGF*β*1 protein level was diminished in the soleus muscle as soon as day 7 after crush in treated muscles.

**Figure 4:**
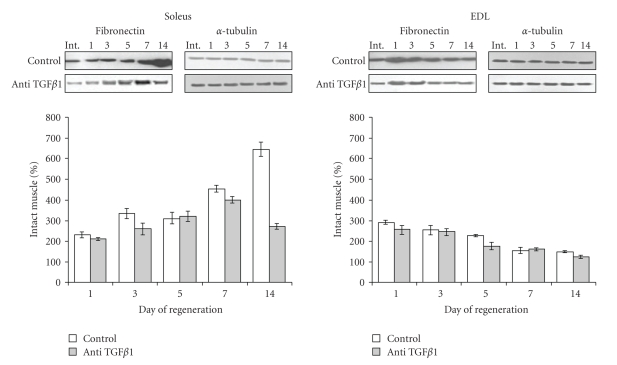
*Effect of anti-TGF* *β* *1 on fibronectin protein level during soleus and EDL muscle regeneration*. Densitometric analysis of immunoblots performed on extracts from control untreated or anti-TGF*β*1 treated soleus and EDL muscles at the indicated days after crush. Level found in the corresponding intact muscle was taken as reference (100%). Results are mean ± SE of 3 independent determinations each of them including 3 animals. Representative immunoblots of fibronectin from control and treated soleus and EDL muscles are also shown. *α*-tubulin was used as control of loading.

The reduction of TGF*β*1 protein level in the presence of anti-TGF*β*1 correlated with improvement of the muscle morphology shown by histology (Figure 1). To further evaluate the impact of TGF*β*1 impairment on fibrosis in the muscle tissue, fibronectin protein expression was examined in both muscle types in the course of regeneration. This protein of the extracellular matrix is indeed widely used as a marker of tissue fibrosis, in particular in skeletal muscle where it is part of endomysium and perimysium [49]. The level of fibronectin in untreated soleus regenerating muscle (control) at day 1 after crush was about 2-3 times higher than in an intact muscle taken as a reference (100%) (Figure 4). Then, fibronectin protein level further increased in regenerating soleus muscle, reaching its highest level at day 14 (644%) after crush. At this time, fibrosis was clearly visible on histological pictures. In EDL muscles which spontaneously regenerate correctly without any treatment after injury in this model, fibronectin protein increased about 3 times at day 1 after crush but decreased to almost intact level at day 15 after crush. Treatment of EDL regenerating muscle with anti-TGF*β*1 antibody slightly decreased the level of fibronectin protein. In contrast, in soleus muscle anti-TGF*β*1 antibody highly decreased the level of fibronectin protein especially at day 14 after crush. These results illustrated a beneficial role of anti-TGF*β*1 to reduce fibrosis during soleus muscle regeneration.

### 3.3. Properties of Myoblasts Isolated from Soleus and EDL Muscles

Immunohistological studies have shown that TGF*β*1 was found around muscle fibers in intact muscles, some cells at the periphery of fibers being highly labelled. In regenerating muscles, the antibody against TGF*β*1 decorated small cellular structures, presumably activated satellite cells or small myotubes (not shown). This suggested that the TGF*β*1 found in regenerating muscles, especially in soleus type muscle, might be attributable not only to fibroblasts or infiltrating inflammatory cells [50] but also to resident myogenic cells. Therefore, we investigated whether differences between fast and slow type muscles, especially as concerns TGF*β*1 protein level, could result, at least in part, from intrinsic properties of myoblast cells.

**Figure 5:**
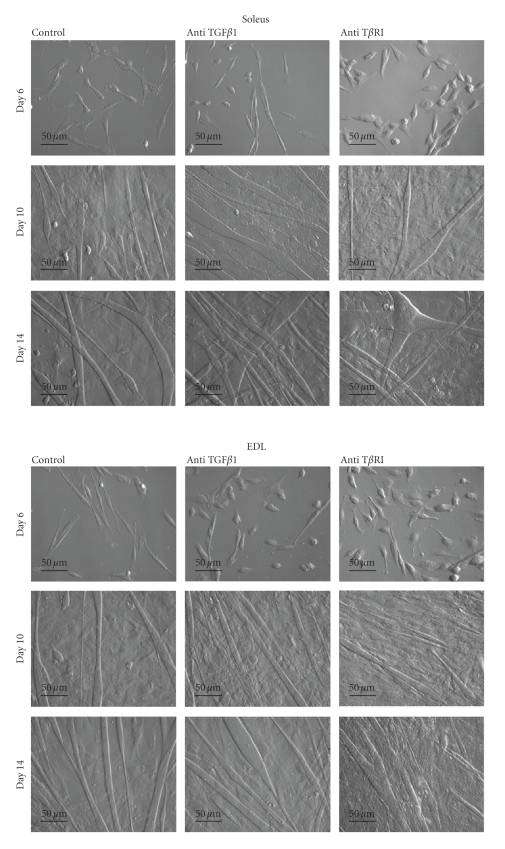
*Morphological aspects of primary cultures of myoblasts isolated from soleus and EDL muscles*. Untreated myoblasts (control) or myoblasts treated at day 4 with either anti-TGF*β*1 or anti-T*β*RI antibody were cultured as described in the Materials and Methods Section. Observations were performed at the indicated days after plating using an Hoffman contrast objective. Bars: 50 *μ*m.

**Figure 6:**
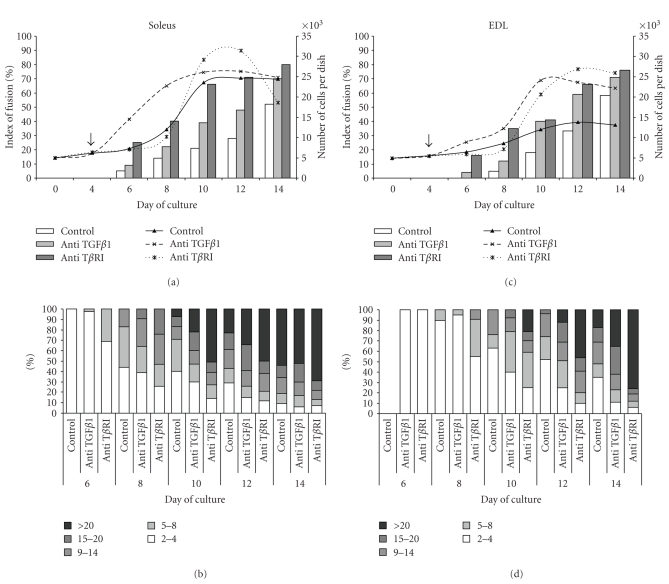
*Growth and differentiation of soleus and EDL derived myoblasts*. *Effects of anti-TGF* *β* *1 and anti-T* *β* *RI antibody treatments*. Untreated myoblasts (control) and myoblasts treated at day 4 after plating (arrow) with either anti-TGF*β*1 or anti-T*β*RI antibody were cultured as described above. In the cultures of (a) soleus and (c) EDL derived myoblasts, cellular growth (shown as curves) was measured by counting the cells after trypsin dissociation. Index of fusion (shown as bars) was expressed as the percentage of nuclei found in myotubes compared to the total number of nuclei. Distributions of myotubes (as % of total myotubes) found in (b) soleus and (d) EDL derived myoblast cultures according to their size, depending on the number of nuclei they contained. Counting was performed at the indicated days after plating on cultures stained with May-Grünwald-Giemsa. Values are mean ± SE of three independent cultures.

Patterns of proliferation and differentiation of myoblasts isolated from soleus and EDL muscles were established at first. Morphological aspects of these cultures are shown on Figure 5. Under our culture conditions, where a high concentration of serum was permanently used (10% FBS and 10% horse serum), the number of cells began to increase after the 4th day following plating. It increased continuously up to day 10 in soleus (Figure 6(a)) and EDL (Figure 6(c)) cell cultures when it reached a plateau. However, this plateau in soleus cultures at day 10 was about 50% higher than in EDL cultures. Spontaneous differentiation in cultures did not begin prior to the 6th day after plating. In soleus myoblast control cultures (Figures 6(a) and 6(b)), the first myotubes appeared at day 6, thus earlier than in the EDL myoblast cultures (Figures 6(c) and 6(d)), and the myotubes formed were bigger than in the EDL cells. For instance, at day 10, myotubes containing more than 20 nuclei were seen in soleus cell cultures whereas the biggest myotubes seen in EDL cell cultures contained only up to 14 nuclei. But finally, at day 14 after plating, fusion index reached about 55% in both types of myoblast cultures. This showed that soleus myoblasts differentiated more precociously than EDL myoblasts.

### 3.4. Impairment of TGF*β*1 Activity by Anti-TGF*β*1 or Anti-TGF*β* Receptor I (T*β*RI) Antibodies Increased Proliferation and Accelerated Myoblast Fusion

Treatments of cultures from day 4 with anti-TGF*β* resulted in an increased myoblast growth and accelerated myotube formation in myoblasts isolated from both soleus (Figures 6(a) and 6(b)) or EDL muscles (Figures 6(c) and 6(d)). The stimulation of proliferation were more striking in the case of soleus myoblast cultures especially between days 6 and 10, but finally the total number of cells was not increased at day 14 compared to untreated controls. In EDL cell cultures, the rate of proliferation was highly increased after day 8 (4 days of treatment), and finally at day 14, there were about 60% more cells in treated cultures. This suggested that TGF*β*1 behaves as an inhibitor of myoblast growth and that impairment of its activity increases cell proliferation.

Compared to control, anti-TGF*β*1 antibody also highly accelerated myoblast fusion as shown on Figure 5. The index of fusion (Figure 6) was increased in both soleus and EDL myoblasts and reached about 65 to 70% at day 14 (Figures 6(a) and 6(c)). In anti-TGF*β*1 antibody-treated cultures, fusion of soleus myoblasts began earlier than in EDL cell cultures, and the sizes of myotubes was more important (Figures 6(b) and 6(d)).

Treatments of myoblasts with anti-T*β*RI antibody also increased cell growth, but this increase was delayed by about 2 days compared to anti-TGF*β*1 antibody treatment. Anti-T*β*RI prolonged cell growth in both soleus and EDL myoblast cultures after day 10 following plating. Interestingly, anti-T*β*RI accelerated myoblast fusion and increased the size of myotubes even more efficiently than anti-TGF*β*1 (Figure 6).

Taken together, these results have shown that neutralization of TGF*β*1 activity or signalling through TGF*β*RI impairment induced an increase in both proliferation and fusion of myoblasts. Since the effects of neutralization of TGF*β* activity on proliferation were kinetically different in soleus and EDL myoblasts cultures, this suggested also that these cultures differed in the pattern of expression of TGF*β* *1* in the course of differentiation.

### 3.5. Patterns of TGF*β*1 and T*β*RI Protein Expression during In Vitro Differentiation of Soleus and EDL Derived Myoblasts

**Figure 7:**
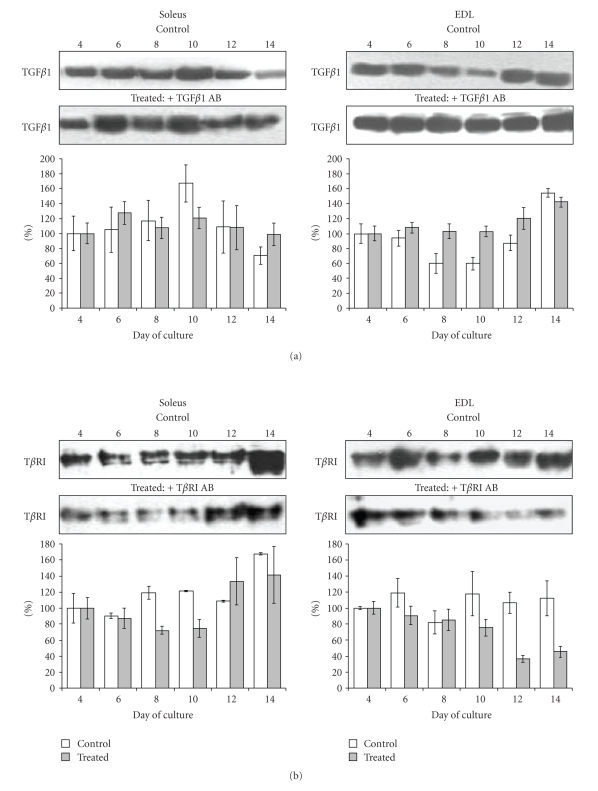
*Effects of anti-TGF*β*1 and anti-T*β*RI antibody treatments on TGF* *β* *1 and T* *β* *RI protein levels in soleus and EDL derived myoblast cultures*. Representative Immunoblots of (a) TGF*β*1 or (b) T*β*RI from cultures of myoblasts derived from soleus and EDL muscles treated with anti-TGF*β*1 and anti-T*β*RI, respectively. Band densities of membranes were scanned. Results are given as bars (white for control and gray for treated cultures) as a percentage of the density is found at day 4 in each condition. Each experiment was performed 3 times and the results were expressed as mean ± SE. Control of loading with *α*-tubulin content in the immunoblot was routinely checked and did not display significant variability upon samples (not shown).

Immunoblot analyses of TGF*β*1 protein content were performed from day 4 of culture in both soleus and EDL derived myoblasts. The amount of TGF*β*1 found at day 4 was taken as reference (100%). In untreated (control) soleus myoblast cultures (Figure 7(a)), the level of TGF*β*1 protein in cellular extracts increased to peak at day 10 after plating. In contrast, in untreated EDL myoblast cultures TGF*β*1 protein level slightly decreased at day 8 compared to day 4, and then it increased to reach a level about 160% of the day 4 level at day 14 of culture. Treatments with anti-TGF*β*1 antibody seemed to attenuate the variations of TGF*β*1 amounts in the course of differentiation, in both soleus and EDL myoblast cultures during (Figure 7(a)).

The patterns of T*β*R1 expression in soleus or EDL control myoblast cultures also differed slightly (Figure 7(b)). Whereas it did not change markedly during the 2 weeks of culturing in control EDL cells, it increased slightly at day 14 in control soleus cell cultures. Treatment of myoblasts with anti-T*β*RI antibody resulted in modification of T*β*RI protein level that dropped of about 30% at days 8 and 10 in soleus cultures before increasing again. In EDL treated cultures, the T*β*RI receptor level dropped at day 12 after plating, thus 4 days later than in soleus cell cultures.

These results have shown that myoblasts from soleus and EDL muscles express TGF*β*1 in vitro and display a differential pattern of TGF*β*1 and T*β*RI protein expressions when differentiating under identical cell culture conditions. Changes in patterns of these proteins were all delayed by about 2 to 4 days in EDL cultures compared to soleus cultures.

### 3.6. The GAG Pattern Change during Soleus and EDL Muscle Regeneration

**Figure 8:**
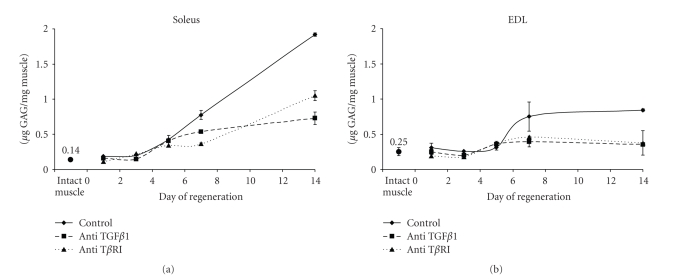
*Sulphated GAGs content during soleus and EDL muscle regeneration*. Total sulphated GAGs were measured using a method based on dimethylmethylene blue coprecipitation with GAGs (see Materials and Methods Section) in extracts from regenerating control treated with the indicated antibodies. Results are expressed as *μ*g of GAG per mg of muscle fresh weight ± SE. Results are means from 3 animals repeated 3 times.

GAGs are known to contribute to changes in cell behavior by modifying the storage and availability of bioactive molecules such as TGF*β*. Thus the pattern of GAGs at the beginning of regenerating soleus and EDL muscle was established (Figure 8). In both muscles, sulphated GAG content increased compared to intact muscles during the first two weeks of the repair process. Whereas it reached a plateau at day 7 after crush in EDL muscle, it increased continuously in soleus regenerating muscle up to day 14. Treatment of muscles with the anti-TGF*β*1 antibody highly reduced the amounts of GAGs in both muscles from day 5 after crush, especially in EDL. When crushed muscles were injected with anti-T*β*RI antibody, GAG amounts in both muscles were reduced almost as efficiently as in the presence of anti-TGF*β*1 (Figure 8). Thus a prolonged presence of TGF*β*1 in muscle was associated with sustained GAG production, especially in soleus muscle. This is in support of the hypothesis that TGF*β*1 activity alters GAG amount during muscle regeneration as in number of other studies.

Skeletal muscles are composed of many different cell species, including inflammatory cells, each of them diversely contributing to the production of GAGs. It was therefore of interest to analyze the GAGs produced by the cells responsible for muscle repair, that is, myoblasts and to examine the effect of TGF*β*1 neutralization on these GAGs.

### 3.7. Changes in GAG Pattern during Differentiation of Soleus and EDL Derived Myoblasts

**Figure 9:**
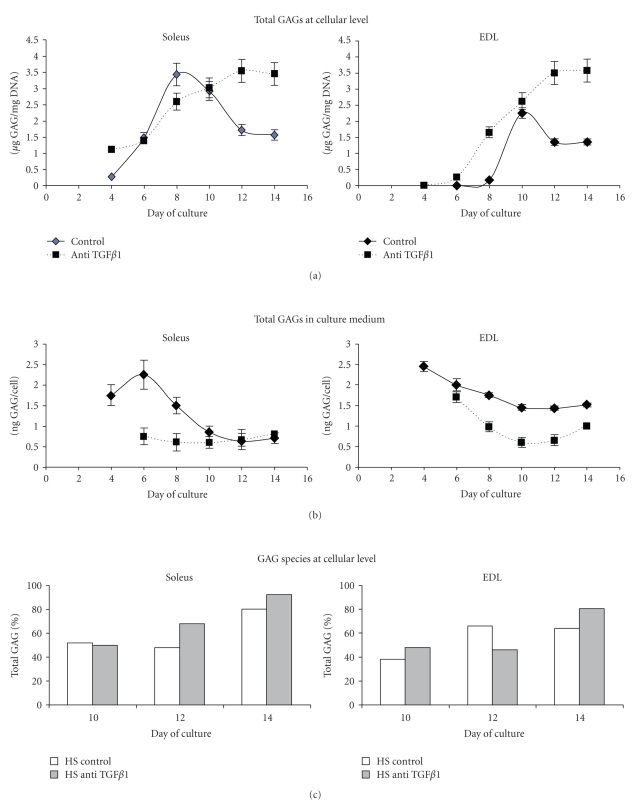
*Sulphated GAG levels produced by differentiating myoblasts isolated from soleus and EDL muscles*. Untreated myoblasts (control) or myoblast treated at day 4 with anti-TGF*β*1 antibody were cultured as described in Materials and Methods. (a) Total sulphated GAGs at cellular level at the indicated days of culture. Data are mean ± SE from 3 determinations. (b) Sulphated GAG levels in conditioned medium of soleus and EDL derived myoblast cultures. Results were expressed as ng of GAG produced per cell ± SE as a result of 3 determinations. (c) Analysis of GAG species in EDL and Soleus derived myoblasts. HS, expressed as % of total GAG, was determined after a chondroïtinase ABC treatment of the total GAG samples. Two determinations, that did not differ by more than 10%, were used in this experiment.

The pattern of total GAG content at cellular level slightly differed in Soleus and EDL derived myoblasts. In soleus myoblasts, the produced GAGs increased from day 4 to peak when myotubes were formed at day 8 and reached 3.5 *μ*g of GAG/*μ*g DNA then, they decreased slightly (Figure 9(a)). The GAG peak in EDL derived myoblasts occurred 2 days later than in soleus cells and was at its maximum at day 10 when it reached 2.5 *μ*g GAG/*μ*g DNA (Figure 9(a)). In fact, GAGs produced were almost exclusively found in culture medium when myoblasts proliferate (day 4). GAGs in culture medium then diminished when cells differentiated (Figure 9(b)), more drastically in the case of soleus cell culture. The amount of HS species proportionally increased when cells of both types achieved their differentiation, especially in soleus cell cultures (Figure 9(c)).

Treatment of cells with anti-TGF*β*1 altered the total GAG content. In both types of myoblasts, it induced a sustained level of total GAGs at the cellular level between days 10 and 14 of culture in differentiating cells when GAGs diminished in controls. At the same time, anti-TGF*β*1 highly reduced the amount of GAGs found in culture medium (Figure 9(b)). Anti-T*β*RI had similar effects than anti-TGF*β*1 on GAG distribution between cells and medium (not shown). The treatment of cells with anti-TGF*β*1 further increased the proportion of HS compared to controls in both cultures (Figure 9(c)).

These results have shown that soleus and EDL derived myoblasts synthesize GAGs that accumulate at the cellular level when myoblasts fuse into myotubes. This accumulation took place earlier in soleus than in EDL muscle cell cultures but occurred before the peak of TGF*β*1 in both muscles. The antibodies that impaired TGF*β*1 action accelerated myotube formation. It also accelerated the accumulation of GAGs at cellular level where the augmentation of HS species was emphasized.

## 4. Discussion

Slow and fast twitch muscles, soleus, and EDL, respectively, displayed differential aspects in the course of their regeneration after crush. As previously observed [14], whereas EDL muscle regenerates properly, the soleus muscle displays fibrosis. The hypothesis that was presently explored was that the differential regeneration ability of the fast and slow muscles was associated to differential expressions of TGF*β*1 in these muscles and in myoblasts derived from them.

The first observation of this study was indeed that soleus and EDL regenerating muscles displayed different patterns of TGF*β*1 protein amounts as shown by immunoblot and ELISA techniques. After an initial rise of TGF*β*1 at day 1 after crush, the soleus showed a sustained level of this protein over the 14 days after injury, but the amount of TGF*β*1 found in EDL diminished at day 7. Immunoneutralization of TGF*β*1 action by using antibodies improved muscle regeneration as shown by histological analysis. Correlatively, anti-TGF*β*1 treatment also anticipated a drop in the level of this cytokine detected by immunoblotting, especially in soleus regenerating muscles.

In many studies, TGF*β*1 appears as a key component that regulates a fibrotic loop in muscle cells. In physiological condition, it is commonly admitted that synthesis of extracellular matrix molecules such as fibronectin increases during repair of tissue but decreases with the formation of normal parenchyma. In our crush induced regeneration model, EDL muscle which regenerates correctly, displayed such a transient increase in TGF*β*1 and fibronectin. That was not the case in regenerating soleus muscle where sustained TGF*β*1 and fibronectin protein levels were correlated to a poor quality of regeneration. Li at al. have shown that TGF*β*1 triggers a transformation of myoblasts to fibroblasts [23]. In addition, Brandan's group have recently shown that PG such as decorin and biglycan, which are known to regulate TGF*β*1 bioavailability, might originate at least partially from fibroblasts in vivo [49]. The improvement of the quality of regenerated soleus muscles observed here in the presence of anti-TGF*β*1 might come from the fact that this loop was minimized by treatment with the antibodies that impaired TGF*β*1 effect.

The possibility that differentiating myoblasts in regenerating muscle might participate to the TGF*β*1 production had to be evaluated. To our knowledge, the production of TGF*β*1 by myoblasts isolated from soleus and EDL muscles and grown in primary cultures has never been established. It has been shown here that both cultures expressed TGF*β*1 but the patterns of TGF*β*1 expression differed between the two types of cultures. When TGF*β*1 protein increased in soleus cell cultures at day 10, it increased later (days 12–14) in EDL cell cultures. In addition, myoblasts derived from these muscles differ in proliferating and differentiating properties, thus corroborating previous findings [16]. It was presently shown that in soleus cell cultures some myoblasts fused into myotubes earlier than in EDL cultures, while other myoblasts remained proliferating a longer time than in EDL cultures.

Conflicting reports have been made on the role of TGF*β* in skeletal myoblast differentiation and in vivo myogenesis [51]. Although many cells respond equally to different isoforms, it has been shown that TGF*β*1 isoform totally inhibits the terminal differentiation of the Sol 8 cell line [52]. In the present study it was seen that TGF*β*1 produced by cells did not prevent myoblast to fuse, whatever was its amount. However, although cell treatment with neutralizing antibodies did not alter extensively the apparent amount of TGF*β*1 protein detected by immuno electrophoresis, it has an important effect on cell behavior. It seemed to induce an appropriate state in cells that triggers them to fuse more precociously and intensively. The anti-T*β*RI antibody was even more efficient than the anti-TGF*β*1 in triggering myoblast fusion. The presence of anti-T*β*RI antibody reduced for several days, in both cultures, the amount of receptor, suggestive of an autocrine loop between TGF*β*1 and its receptor. Precocious fusions observed here in treated cultures could not be due to an increasing cell density that would enhance the probability of fusion between neighbouring myoblasts. In fact, at similarly low cellular densities, some cultures presented numerous myotubes and others not. Rather, these precocious fusions reflected intrinsic cellular properties. As suggested by a study of Brandan's group [53], the increase in fusion associated to TGF*β*1 impairment by an appropriate antibody could be linked to an enhanced ability of myoblasts to migrate.

Other factors might be involved in regulating the cytokine activity. TGF*β* family members are known to have affinity to proteoglycans. Most of the studies concerning the role of proteoglycans on the regulation of TGF*β* activity have focused on the expression of their core protein components. But a growing body of evidence has attributed to sulphated glycosaminoglycans, a major importance in regulating growth factor, cytokine, and chemiokine effects including TGF*β*1. In the present study, the amount of sulphated GAGs were increased in regenerating muscles within the first 2 weeks post-injury, especially in the soleus. These GAGs might contribute to control TGF*β*1 activity. Part of these GAGs were produced by activated satellite cells. It is shown here that soleus and EDL derived myoblasts produced increasing amounts of GAGs in vitro when cells began to fuse. At the same time, the amount of GAGs in the culture medium decreased. The peak of GAGs occurred two days earlier in soleus myoblast cultures compared to EDL myoblast cultures. This finding could be correlated with the observation that soleus myoblasts fused earlier than EDL myoblasts. It must be emphasized that the maximum of GAG production at cellular level occurred before the maximum of TGF*β*1 protein in both culture types. These GAGs might participate to the control of TGF*β*1 signaling.

We previously have shown that an increasing amount of GAGs, especially of the HS species, could be associated to differentiating myoblasts using the C2.7 myoblast cell line (Barbosa et al. 2005). An increased HS production by differentiating soleus and EDL myoblasts was also observed here. It was also shown that the attenuation of TGF*β*1 activity by using antibodies increased the amount of GAGs produced by myoblasts at the cellular level and accelerated the shift of GAG composition from CS to HS species. As it has been shown that DS/CS moiety of some proteoglycans sequesters TGF*β* [54] and that its biological activity could be negatively regulated by HS [55], it can be conceivable that GAGs, principally of HS species synthesized by differentiating cells, would finally diminish the action of this growth factor at cellular level. The presence of antibodies would amplify this effect by favoring HS species accumulation. Such a virtuous loop has been suggested in a study where epithelial cells were used. In these cells, deficiency or enzymatic removal of HS at the cellular surface attenuates degradation of TGF*β*1 and increases TGF*β*1 signaling [55]. However, since in vivo in the presence of the antibodies, total amount of GAGs measured in muscles was reduced compared to untreated muscles in contrast with what was observed in vitro at myoblast level. Thus, the participation of myoblasts in vivo in the production of GAGs might contribute to a minor degree compared to the other cells, including inflammatory cells and endothelial cells that compose the muscular tissue, without forgetting fibroblasts as recently underscore by Brandan's group [56].

The present study indicated that the control of TGF*β* activity is necessary to improve regeneration of injured muscle and myoblast differentiation, and that this effect is in part related to GAG composition of muscle cell environment. The next steps would be to decipher the sequence of GAGs produced in vivo in relation to interacting bioactive proteins including TGF*β*1 in regenerating muscles and to determine their spatial distribution. Routine GAG sequencing techniques, as well as tools for analyzing localization of sequences biologically relevant for muscle differentiation, remain to be available. Nevertheless, the present study supports the idea that antagonizing TGF*β*1 action with neutralizing antibody could rescue accurate skeletal muscle regeneration in part by acting at the myoblast level through an alteration of GAG environment.
